# Comparison of Physicochemical Properties and Immunomodulatory Activity of Polysaccharides from Fresh and Dried Litchi Pulp

**DOI:** 10.3390/molecules19043909

**Published:** 2014-03-31

**Authors:** Fei Huang, Ruifen Zhang, Yang Yi, Xiaojun Tang, Mingwei Zhang, Dongxiao Su, Yuanyuan Deng, Zhencheng Wei

**Affiliations:** 1 Department of Food Science and Technology, Huazhong Agricultural University, Wuhan 430070, China; 2 Sericultural and Agri-food Research Institute, Guangdong Academy of Agricultural Sciences, Guangzhou 510610, China; 3 College of Food Science & Engineering, Wuhan Polytechnic University, Wuhan 430023, China

**Keywords:** litchi, drying, polysaccharides, anti-tumor activity, immunomodulatory activity

## Abstract

Drying is commonly used for preservation and processing of litchi. However, its polysaccharide structure may be altered by the drying process, resulting in biological activity changes. Polysaccharides from fresh and dried litchi pulp (denoted as LPF and LPD, respectively) were isolated, investigated by GC-MS, GPC and UV/IR spectrum analysis and their antitumor and immunomodulatory activities were evaluated *in vitro*. LPD, the molecular weight of which was lower than that of LPF, contained more protein, uronic acid, arabinose, galactose and xylose. Compared with LPF, LPD exhibited a higher inhibitory effect on the proliferation of HepG2, Hela and A549 cells from 50–750 μg/mL. LPD was also a better stimulator of spleen lymphocyte proliferation, NK cells cytotoxicity and macrophage phagocytosis from 50–400 μg/mL. In summary, drying could change the physicochemical properties and enhance the bioactivity of polysaccharides from litchi pulp. This finding is supported by the fact that dried litchi pulps are used in Traditional Chinese Medicine.

## 1. Introduction

Litchi (*Litchi chinensis* Sonn*.*), originating from China, is widely planted in warm climates around the World. Because of its attractive appearance and delicious flavor, litchi is becoming increasingly popular. Besides providing sensory satisfaction, litchi fruit extracts have been reported to have antioxidant properties [1,2], proinflammatory effects [3], anticancer activity [4], anti-apoptotic activities [5] and immune-stimulating activities [6]. Many bioactive compounds have been identified from litchi pulp, including polysaccharides which exhibit antioxidant activities [7]. 

Litchi is a seasonal and highly perishable fruit that is commonly dried for long term storage and further processing. However, the drying process has been shown to have a significant effect on the physicochemical properties and characteristic components of the material. A previous study showed that drying changed the content of dietary fiber in orange by-products [8], as well as the content of total phenolics, flavonoids and lycopene in tomatoes [9]. Furthermore, drying may cause irreversible modifications to the chemical composition and structure of polysaccharides. For example, a sun-drying process caused the modification of monosaccharide composition and FT-IR characteristics of polysaccharides from pear pulp [10]. The molecular size distribution and proportions of (1→3,6)-linked galactose residues of pectin polysaccharides from Japanese persimmon fruit were also changed during the sun-drying process [11]. 

It is well known that the bioactivities of native polysaccharides are closely related to their chemical composition and structure. The crude polysaccharides from the dried fruit body of *Lentinus polychrous Lév* contained more proteins, total polysaccharides and reducing sugar and showed stronger antioxidant activity and cytotoxicity against MCF-7 tumor cells [12] than those from fresh fruit bodies. Furthermore, thermal drying could change the structure of mushroom polysaccharides and result in aggregation of polysaccharides due to removal of the hydration layer. The aggregated polysaccharides were larger, but had lower antioxidant activity compared with polysaccharides from freeze dried material [13]. Although the structure and activity of litchi polysaccharides have been reported in previous research [1,7,14,15], little is known about the effects of drying on the polysaccharides in litchi. Therefore, the polysaccharides from fresh and hot-air dried litchi pulp were analyzed in the present study. The objectives were: (1) to reveal the differences in the physicochemical properties of the polysaccharides from fresh and hot-air dried litchi pulp; (2) to compare their antitumor and immunomodulatory activity *in vitro*; (3) to elucidate the relationships between their structure and bioactivity.

## 2. Results and Discussion

### 2.1. Preliminary Characterization of Litchi Polysaccharides

#### 2.1.1. The Chemical Compositions of Litchi Polysaccharides

Two hundred grams fresh pulp and 50 g dried pulp of litchi were used to obtain litchi polysaccharides. The yield of polysaccharides was 1.15% from fresh litchi pulp and that from dried litchi pulp was 3.58%. The amount of neutral sugar, uronic acid and protein, as well as molecular weight distribution and monosaccharide composition of LPF and LPD are summarized in Table 1. Obviously, LPD contained more uronic acid and binding protein than LPF (*p* < 0.05). The result was consistent with Thetsrimuang’s report in which the protein content of polysaccharides from dried *Lentinus polychrous Lév* was significantly higher than in fresh ones [12]. The difference in protein content of LPF and LPD could be due to thermal effects which could induce conformational changes in the polysaccharides and proteins that may result in the formation of protein-polysaccharide complexes by electrostatic interactions [16], since free but not binding protein could be removed from the polysaccharides by the Sevag method [17]. Moreover, Asgar reported the uronic acid content in polysaccharides from Japanese persimmon fruit increased after sun-drying compared with fresh material [11], which was consistent with our results.

**Table 1:** The chemical compositions of litchi polysaccharides LPF and LPD.

Samples	LPF	LPD
Neutral sugar (W%)	66.85 ± 1.12	65.73 ± 1.76
Uronic acid (W%)	2.09 ± 0.48	4.72 ± 0.27 *
Protein (W%)	3.94 ± 0.16	6.48 ± 0.24 *
Molecular weight (Da)	970085	370365; 8207
Monosaccharide composition (%)		
Rhamnose	0.31	1.86
Arabinose	5.44	17.62
Xylose	0.71	3.21
Mannose	15.18	10.56
Glucose	66.1	20.82
Galactose	11.58	41.18

* *p* < 0.05.

LPF had a large molecular weight (M_w_) of 970,085 Da, while LPD was observed to be composed of two components with relatively small molecular weights (Table 1). The results were similar to the change of molecular weight of polysaccharides from *Inonotus obliquus* which decreased with thermal treatment [18]. It was inferred that the cleavage of the polysaccharides chain accounted for the decrease of M_w_ value upon thermal treatment.

LPF and LPD had the same monosaccharide composition, but their monosaccharide molar ratios had apparent differences. Compared with LPF, the molar ratio of glucose in LPD exhibited a 45.28% decrease, while galactose and arabinose were increased by 29.6% and 12.18%, respectively. Mirhosseini *et al.* reported freeze-drying and spray-drying processes resulted in the significant reduction of glucose, galactose and arabinose in gum polysaccharides while they were increased by an oven drying process [19]. Asgar *et al.* found the galactose content of pectin polysaccharides decreased while the arabinose content increased with sun-drying [20]. It can be concluded that temperature and method for drying materials are important factors influencing the monosaccharide composition of polysaccharides in them. The differences among our study and others could result from different processing conditions and materials.

The amount of neutral sugar and uronic acid were individually determined by a phenol-sulfuric acid method and an *m*-hydroxydiphenyl method, respectively. The protein content and monosaccharide composition were determined by Bradford’s method and a GC-MS method, respectively. The average molecular weight of the polysaccharides was determined by gel permeation chromatography (GPC). The values for neutral sugar, uronic acid, and protein are expressed as the mean ± SD (*n* = 3). * *p* < 0.05.

#### 2.1.2. UV and IR Spectra Analysis

**Figure 1:**
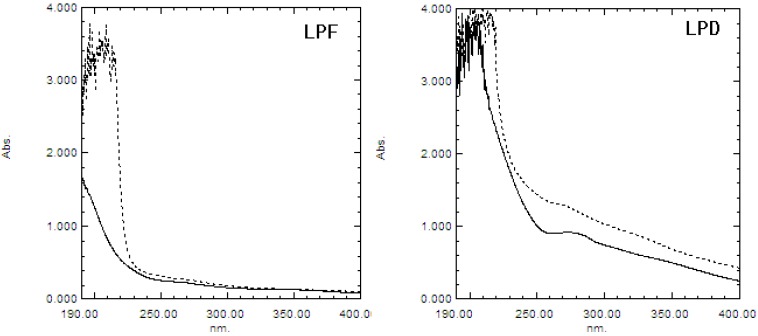
UV spectra of the β-elimination reactions of the litchi polysaccharides LPF and LPD. A dotted line represents the absorption of polysaccharide treated with sodium hydroxide, and a solid line represents the absorption of non-treated polysaccharide.

The UV absorptions of LPF and LPD are shown in Figure 1. The absorption of polysaccharides treated with sodium hydroxide was higher than that of the non-treated control at 240 nm in LPD, but not in LPF, indicating the presence of O-glycosidic bonds in LPD. This may be attributed to the formation of protein-polysaccharide complexes by electrostatic interactions between polysaccharides and proteins with thermal effects [21]. When immersed in a weak alkali solution, the O-glycosidic bonds between serine or threonine and sugars would produce α-aminoacrylic acid and α-aminobutenoic acid, which results in an increase of absorbance at 240 nm [22]. The absorbance at 280 nm in LPD was higher than that in LPF, which indicated the content of protein in LPD was higher than that in LPF, which was consistent with the result obtained from the Bradford determination.

The absorption peaks, functional groups and structural characteristics of LPF and LPD are summarized in Table 2. Both LPF and LPD had hydroxyl groups (3397.4, 3405.6 cm^−1^), alkyl groups (2928.0, 2926.1 cm^−1^) and carboxyl groups (1654.5, 1637.6 cm^−1^), which are characteristic of polysaccharides. In addition, the amino group bands (3397.4, 3405.6; 1654.5, 1637.6 cm^−1^) indicated the presence of protein in LPF and LPD. However, there were some differences between the spectra of LPF and LPD. LPF lacked the absorption peaks at 1541.4 cm^−1^ and 1458.8 cm^−1^ which represented the stretching vibration of C=O, and bending vibration of C-H, respectively. Moreover, the absorption peaks at 1156.0, 917.9 and 848.9 cm^−1^ indicating the ether -C-O-C-, d-glucopyranose ring and α-type glycosidic linkage, respectively, only existed in LPF.

**Table 2:** FTIR spectra of litchi polysaccharides LPF and LPD.

Absorption (cm^−1^) ^a^		Functional group ^b^	Structural characteristics
LPF	LPD		
3397.4	3405.6	hydroxyl group (-OH)	O-H stretching vibration
		amino group (-NH_2_)	N-H stretching vibration
2928.0	2926.1	alkyl group (-CH_2_-)	C-H stretching vibration
1654.5	1637.6	carbonyl group (-C=O or -CHO)	C=O stretching vibration
		amide group (-NH_2_ or –COR)	N-H bending vibration or C=O stretching vibration
		amino group (-NH_2_)	N-H bending vibration
		bound water	
1541.4		amino group (-NH_2_) or amide group (-NH_2_)	N-H bending vibration
		carbonyl group (-C=O)	C=O stretching vibration
1458.8		alkyl group (-CH_2_- or –CH_3_)	C-H bending vibration
1420.0	1420.8	carboxyl group (-COOH)	C-O stretching vibration
1364.0	1375.8	carboxyl group (-COOH)	C=O symmetrical stretching vibration
1270.8	1275.1	carboxyl group (-COOH)	O-H bending vibration
1156.0		ether (-C-O-C-)	C-O stretching vibration
1017.0	1074.7	hydroxyl group (-OH)	O-H bending vibration
917.9		D-glucopyranose ring	Antisymmetrical ring vibration
848.9		α-type glycosidic linkage	C-H bending vibration
764.8	777.4	D-glucopyranose ring	symmetrical ring vibration

^a^ The FTIR spectra of litchi polysaccharides LPF and LPD were determined using a Fourier-transform infrared spectrophotometer over the frequency range of 4,000–400 cm^−1^; ^b^ The functional group and structural characteristics were obtained from [22].

### 2.2. In Vitro Inhibition Effects of Tumor Cell Proliferation of Litchi Polysaccharides

**Figure 2:**
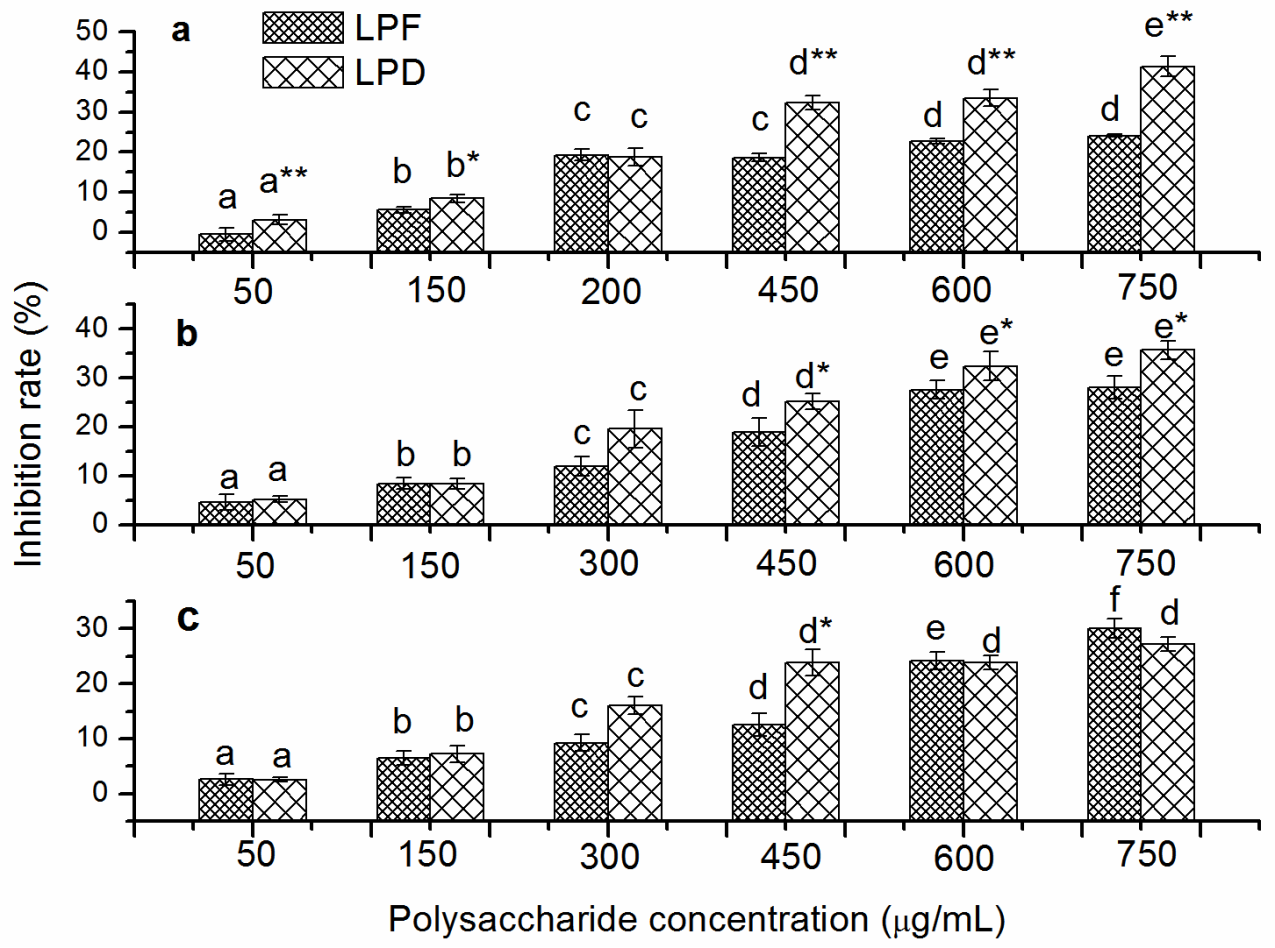
The inhibition of proliferation of cancerous cells by the litchi polysaccharides LPF and LPD. HepG2 (**a**), Hela (**b**), and A549 (**c**) cells were incubated at different concentrations (50, 150, 300, 450, 600 and 750 μg/mL). The percent inhibition were assessed by the methylene blue method and expressed as the mean ± standard deviation (*n* = 6). Bars labeled with different letters represent a statistical difference at *p* < 0.05 among different concentrations of the same sample. (*) 0.01 < *p* < 0.05 and (**) *p* < 0.01 compared with LPF.

The inhibitory activities of litchi polysaccharides on the proliferation of HepG2, Hela, and A549 cells are shown in Figure 2. No cytotoxicity was observed toward three tumor cell lines treated at concentrations ≤ 1000 μg/mL of LPF and LPD (data not shown). Both LPF and LPD inhibited the proliferation of three tumor cell lines in a concentration dependent manner at the tested concentrations. HepG2 cells were inhibited from −0.57% to 24.12% by LPF, while the percent inhibition for LPD ranged from 3.11% at 50 μg/mL to 41.37% at 750 μg/mL (Figure 2a). LPD showed a significantly higher inhibitory effect on the proliferation of HepG2 cells than LPF at the tested concentrations, except 200 μg/mL (*p* < 0.05). Similarly, the proliferation of Hela cells were inhibited 4.61%–28.04% and 5.17%–35.65% by LPF and LPD, respectively, in the dose range of 50–750 μg/mL (Figure 2b). The differences between LPF and LPD in their inhibition of Hela cells growth were exhibited at high concentrations (450, 600 and 750 μg/mL) (*p* < 0.05). Moreover, the growth of A549 cells decreased 2.59%–30.07% and 2.56%–27.17% by the incubation of LPF and LPD at the tested concentrations, respectively (Figure 2c). The differences between LPF and LPD in their inhibition of A549 cells growth were exhibited only at 450 μg/mL (*p* < 0.05).

The anti-tumor cell line activity of crude polysaccharides with free protein was also determined. The results showed that polysaccharides without free protein showed better anti-tumor cell line activities whether for LPF or for LPD (data not shown), which implied that the potential active component was the polysaccharides. The bioactivity of polysaccharides is closely related with their chemical composition and structure. LPD, which had higher uronic acid, showed stronger anti-tumor cell line activity *in vitro* than LPF in this study. It was previously reported that polysaccharides rich in uronic acid exhibited high biological activities because uronic acid residues may alter the properties of polysaccharides and modify their solubility [23,24]. Furthermore, LPD exhibited higher anti-tumor cell line than LPF may due to its molecular weight and monosaccharide composition. Ouchi *et al.* reported that galactomannan was specifically recognized by a galactose receptor on the HepG2 cellular surface, thus polysaccharides rich in galactose had high anti-tumor cell line activities [25]. ASP-3 polysaccharide from *Amomum villosum*, which contained more galactose than did ASP-1 and ASP-2, showed the stronger anti-tumor cell line activity [26].

### 2.3. In Vitro Immunostimulatory Activity of Litchi Polysaccharides

#### 2.3.1. Effects of Litchi Polysaccharides on Splenocyte Proliferation *in Vitro*

**Figure 3:**
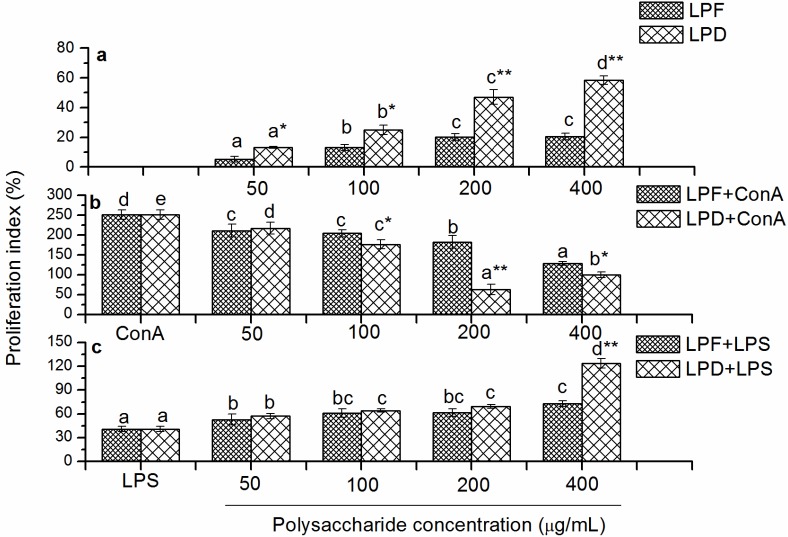
Effects of the litchi polysaccharides LPF and LPD on the proliferation index of splenocytes (**a**), ConA-induced splenocytes (**b**) and LPS-induced splenocytes (**c**) at different concentrations (0, 50, 100, 200 and 400 μg/mL). The proliferation indexes were assessed by the MTT assay and expressed as the mean ± standard deviation (*n* = 6). Bars labeled with different letters represent a statistical difference at *p* < 0.05 among different concentrations of the same sample. (*) 0.01< *p* < 0.05 and (**) *p* < 0.01 compared with LPF.

Lymphocyte proliferation is a crucial event in the activation cascade of both cellular and humoral immune responses. Spleen lymphocyte proliferation induced by ConA *in vitro* was used as a method to evaluate T lymphocyte activity, while that induced by LPS was used to examine B lymphocyte activity [27,28]. The stimulatory effects of LPF and LPD on mouse splenocyte proliferation are presented in Figure 3a. LPF and LPD stimulated the proliferation of mouse splenocyte in a dose-dependent manner. Both LPF and LPD exhibited the highest proliferation indexes (PI) of 20.6% and 58.45% at 400 μg/mL, respectively. LPD exhibited stronger stimulatory activity toward splenocyte proliferation than LPF at the same concentrations (*p* < 0.05).

Moreover, both LPF and LPD showed stimulation toward the proliferation of splenocytes with and without LPS (Figure 3a,c). LPD showed stronger proliferative stimulation than LPF. It was indicated that LPD exhibited stronger stimulation in inducing the proliferation of B lymphocyte than LPF. However, addition of litchi polysaccharides attenuated ConA-induced proliferation of splenocytes (Figure 3b). ConA activated T lymphocyte by binding to the specific receptor on T lymphocyte cell membranes [29]. Litchi polysaccharides may compete with ConA for the specific receptor, which resulted in the attenuation of the ConA-induced splenocytes proliferation. Furthermore, the stronger inhibition of LPD to ConA-induced splenocytes proliferation gave a clue that LPD may show stronger competition for receptor binding than LPF. The results were similar to another report where polysaccharides from the stems of *Ephedra sinica Stapf* exhibited inhibitory effects on the proliferation of ConA-stimulated mouse splenocyte [30]. However, our results were inconsistent with Jing’s report that the purified litchi polysaccharides LCP50W had the potential of promoting the lymphocyte proliferation along with Con A [15]. This discrepancy could, at least partly, be attributed to the different purity of the tested polysaccharides. Crude, not purified polysaccharides were analyzed in this study. In another word, LPD and LPF were composed of different polysaccharide fractions. Therefore, the bioactivity of these crude polysaccharides should be a combined effect of all the fractions. And some fractions unavoidably had different activity from the others.

To date, the effects of polysaccharides from different materials on lymphocyte proliferation were variously reported. *Misgurnus anguillicaudatus* polysaccharides activated T cells via the binding of the polysaccharides to receptors specifically expressed on T cells [31]. Polysaccharides from *Ganoderma lucidum* mediated mouse splenic B cells activation by the induction of Blimp-1, a master regulator capable of triggering the changes of a cascade of gene expression during plasmacytic differentiation [32]. In addition, polysaccharides from evening primrose [27], *Trametes robiniophila Murr* [33] and *Armillaria mellea* [34] exhibited stimulating effects on both ConA and LPS-induced lymphocyte proliferation. The mechanism of which polysaccharides exhibited cell specificity in stimulating lymphocyte proliferation is unclear up till now. The monosaccharide composition and structural characteristic of these polysaccharides were deduced to be important influencing factor.

#### 2.3.2. Effects of Litchi Polysaccharides on NK Cells Cytotoxicity

**Figure 4:**
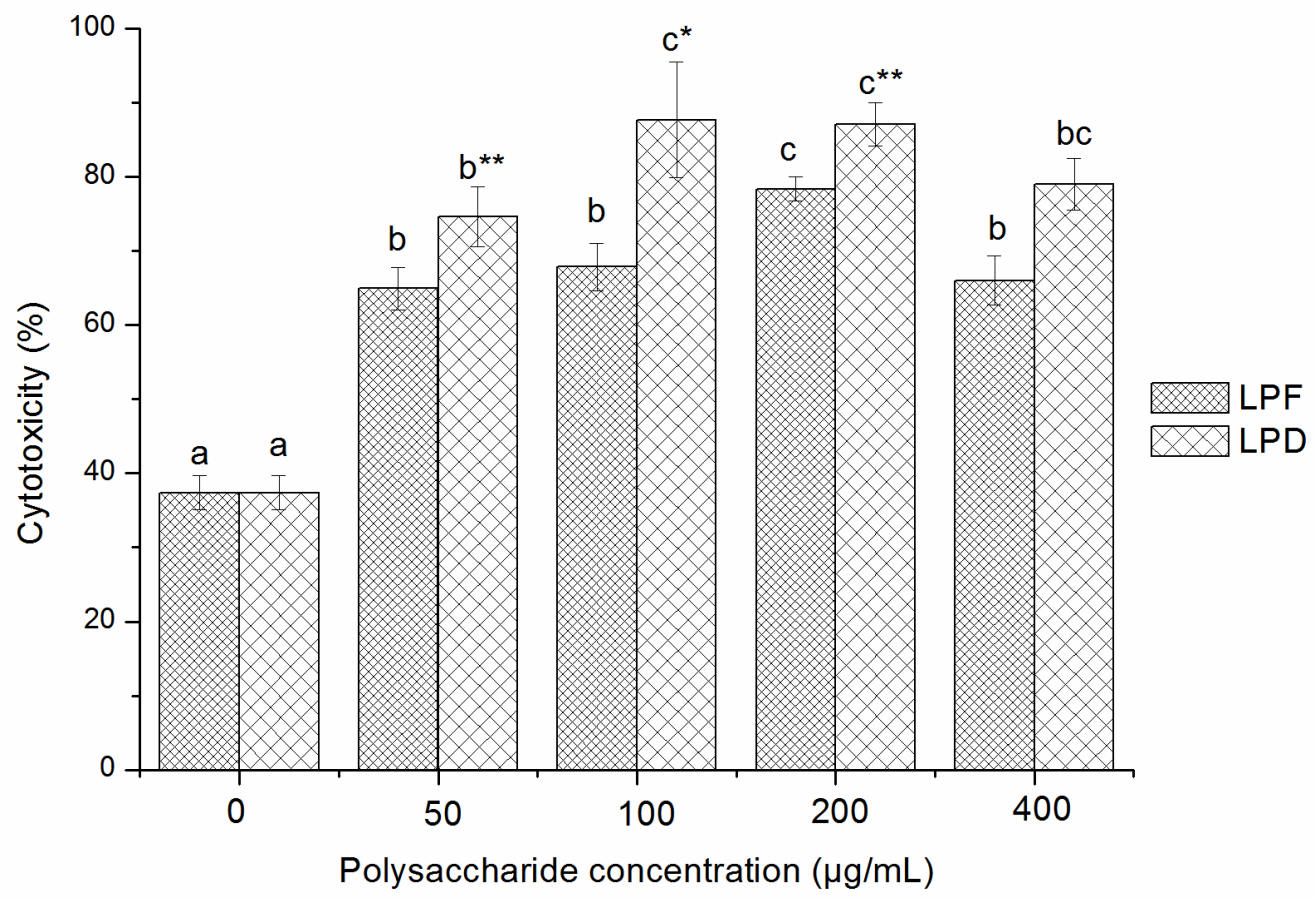
Cytotoxicity of NK cells stimulated by litchi polysaccharides LPF and LPD toward target cells. Cytotoxicity of NK cells was assessed by the MTT assay and expressed as the mean ± standard deviation (*n* = 6). Bars labeled with different letters represent a statistical difference at *p* < 0.05 among different concentrations of the same sample. (*) 0.01 < *p* < 0.05 and (**) *p* < 0.01 compared with LPF.

The effects of LPF and LPD on the cytotoxicity of NK cells against YAC-1 cells are shown in Figure 4. The percent lysis of target cells was 64.95%–78.36% when the splenocytes were stimulated with 50–400 μg/mL LPF and reached a maximum value at 200 μg/mL. The percent lysis of target cells ranged from 74.64% to 87.72% when the splenocytes were stimulated with 50–400 μg/mL LPD, with a maximum value at 100 μg/mL. Furthermore, compared with LPF, LPD showed stronger stimulation on the NK cells cytotoxicity at the same concentrations in the dose range of 50–400 μg/mL (*p* < 0.05).

#### 2.3.3. Effects of Litchi Polysaccharides on Phagocytosis of RAW264.7 Mice Macrophages

**Figure 5:**
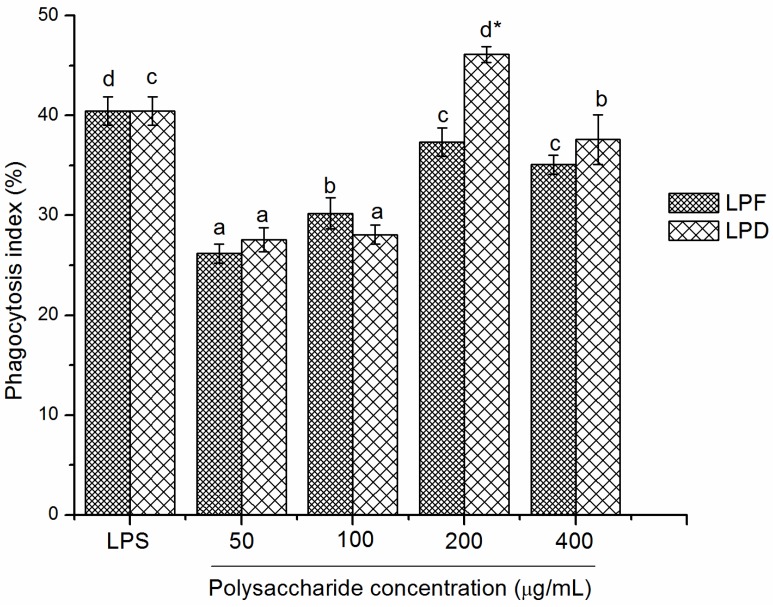
Effects of the litchi polysaccharides LPF and LPD on the phagocytosis of RAW264.7 macrophages. Their phagocytosis indexes were evaluated by measuring the phagocytosis against neutral red and expressed as the mean ± standard deviation (*n* = 6). Bars labeled with different letters represent a statistical difference at *p* < 0.05 among different concentrations of the same sample. (*) 0.01 < *p* < 0.05 and (**) *p* < 0.01 compared with LPF.

The phagocytic activity of RAW264.7 mice macrophages stimulated with LPS or different concentrations of litchi polysaccharides are shown in Figure 5. The phagocytosis indexes induced by LPF were from 26.19% to 37.34% at the concentrations from 50 to 400 μg/mL with a maximum value at 200 μg/mL. In addition, the phagocytosis indexes induced by LPD were in the range of 27.57%–46.12% at the tested concentration range and exhibited the highest at 200 μg/mL. The effect of LPD was significantly better than that of LPF (*p* < 0.05) at 200 μg/mL, but no significant difference was observed at other doses. Compared with LPS (40.76%), 200 μg/mL of LPD (46.12%) exhibited stronger stimulation on phagocytosis of macrophages (*p* < 0.01). 

Polysaccharides bind immune cells via membrane receptors, resulting in stimulation of intracellular signaling cascades for immunologic responses. The stimulating activities of polysaccharides triggered by the recognition of the cell receptors depend on monosaccharide species and monosaccharide content [35]. Lo *et al.* reported that arabinose, mannose, xylose and galactose played an important role in the stimulation of macrophage from *Lentinula edodes* polysaccharides, but not glucose [36]. In addition, mannose played a key role in the immunomodulatory activity of polysaccharide from Longan pulp [28]. The better activities of LPD might be partly related to its monosaccharide proportion and chemical compositions.

## 3. Experimental

### 3.1. Materials and Chemicals

#### 3.1.1. Chemicals and Reagents

Standard dextrans, rhamnose (Rha), arabinose (Ara), glucose (Glc), xylose (Xyl), galactose (Gal), mannose (Man), penicillin–streptomycin solution, concanavalin A (ConA), lipopolysaccharide (LPS) and 3-(4,5-dimethylthiazol-2-yl)-2,5-diphenyltetrazolium bromide (MTT) were purchased from Sigma Chemical Co. (St. Louis, MO, USA). RPMI-1640 medium, new bovine calf serum and Hank’s balanced salt solution (HBSS) were purchased from Gibco Life Technologies (Grand Island, NY, USA). Other regents were analytical grade.

#### 3.1.2. Cells and Animals

Human hepatocellular carcinoma cell line, HepG2; Human uterine cervix carcinoma cell line, Hela; Human lung adenocarcinoma epithelial cell line, A549; YAC-1 mice lymphoma cell line and RAW264.7 mice macrophage cell line were purchased from the Experimental Animal Laboratory of Sun Yat-Sen University (Guangzhou, China). Cells were cultured in RPMI-1640 medium containing 10% fetal calf serum, 100 U/mL penicillin and 100 μg/mL streptomycin at 37 °C and 5% CO_2_.

BALB/c mice (male, 20.0 ± 2.0 g), 6–8 w old were provided by the Experimental Animal Laboratory of Sun Yat-Sen University. The mice were acclimatized for 1 w before being used for the study. All the treatments to animals were performed in accordance with the Guide for the Care and Use of Laboratory Animals.

#### 3.1.3. Preparation of Litchi Fruit

Fresh fruits of litchi (cv. Gui-wei) were offered by Pomology Research Institute of Guangdong Academy of Agricultural Sciences (Guangzhou, China). Ripened fruits were selected based on their uniform size and lack of visual defects. Some of the litchi fruits were dried by intermittent hot air drying with 2.0 m/s flow velocity at 70 °C for 72 h. The intermittent time was 12 h for rewetting, and 12 h for drying time. Dried litchis were peeled and the pulp portion was kept in sealed polyethylene bags at −20 °C for further use. The leftover litchi was used as the fresh fruit for the polysaccharide extraction.

### 3.2. Preparation of Crude Litchi Pulp Polysaccharides

Fresh and dried pulp were cut into small pieces and soaked in 80% ethanol (final concentration in the system) at 4 °C for 24 h, to remove the pigments, monosaccharides and oligosaccharides. After filtration through a Whatman No. 1 paper, the residues were homogenized and then extracted twice with distilled water (1:20, g/mL) at 85 °C for 4 h. The water extracts were filtered, and concentrated to one-fifth of the initial volume in a vacuum evaporator (Eyela, Tokyo, Japan) at 55 °C. The proteins in the extracts were removed using the Sevag reagent [37], and then the polysaccharides were precipitated with four volumes of absolute ethanol for 24 h at 4 °C. The precipitates were collected by centrifugation at 3000 g for 20 min, and washed successively with acetone and petroleum ether. Then the precipitates were lyophilized to obtain fresh and dried litchi pulp polysaccharides, named as LPF and LPD, respectively. The litchi polysaccharides were kept in desiccator at room temperature for further use.

### 3.3. Preliminary Characterization of Litchi Polysaccharides

#### 3.3.1. Analysis of the Chemical Characteristics of Litchi Polysaccharides

Neutral sugar content was determined by the phenol-sulfuric method using glucose as the standard [38]. The protein content was measured according to Bradford’s method with a standard curve using bovine serum albumin (BSA) [39]. The uronic acid content was determined by the *m*-hydroxydiphenyl method with d-glucuronic acid as the standard [40].

The monosaccharide composition of the litchi polysaccharides were determined by GC-MS. Briefly, polysaccharide samples (40 mg) were dissolved in 2 mol/L H_2_SO_4_ (10 mL) and hydrolyzed at 100 °C for 6 h. After neutralizing the residual acid with BaCO_3_, the hydrolysate was filtered through 0.2 μm syringe filters (Whatman, Sanford, ME, UK) and dried under a stream of N_2_, then dissolved in pyridine (5 mL) with hydroxylamine (70 mg) at 90 °C for 30 min. The samples were cooled and acetic anhydride (1 mL) was added and the mixture was incubated at 90 °C for 30 min. The acetylated hydrolysate was extracted by trichloromethane, following by evaporation under a stream of N_2_. The final product was analyzed by GC-MS, using an Agilent 6890 GC instrument (Agilent Technologies Co., Ltd., Colorado Springs, CO, USA) equipped with a DB-1 column and an Agilent 5973 MS detector. Six monosaccharides (arabinose, mannose, rhamnose, galactose, xylose, and glucose) were used as the external standards to identify the composition of the polysaccharides.

#### 3.3.2. Determination of Molecular Weights of Litchi Polysaccharides

The average molecular weights of the litchi polysaccharides were determined by gel permeation chromatography (GPC), which was performed on a Sephacryl S-300HR column (1.6 × 70 cm). Standard dextran including T-4 (molecular mass, 4 × 10^3^ Da), T-10 (1 × 10^4^ Da), T-40 (4 × 10^4^ Da), T-70 (7 × 10^4^ Da), T-500 (5 × 10^5^ Da), and T-2000 (2 × 10^6^ Da) were used as molecular mass markers.

#### 3.3.3. UV and IR Spectra Analysis

β-Elimination reaction: The samples were dissolved in distilled water and forced through a 0.45 μm filter membrane to obtain a 1 mg/mL polysaccharide solution. The solution was mixed with an equal volume of distilled water or 0.4 mol/L NaOH. After 1.5 h incubation at 45 °C, the mixture was then scanned in the wavelength range of 190–400 nm using a UV spectrophotometer (UV 1800, Shimadzu, Kyoto, Japan).

Infrared spectral: The IR spectra of the polysaccharides were determined using a Fourier-transform infrared spectrophotometer (FTIR) (Nexus 5DXC FT-IR, Thermo Nicolet, Austin, TX, USA). The samples were mixed with potassium bromide (KBr) powder and then pressed into 1 mm thick pellets for FTIR measurement in the frequency range of 4,000–400 cm^−1^.

### 3.4. In Vitro Inhibition Assay of Tumor Cells Proliferation of Litchi Polysaccharides

#### 3.4.1. Cytotoxicity Assay of Tumor Cells

The cytotoxicity of litchi polysaccharides was measured by using a methylene blue assay method [41]. Briefly, when each cancer cell line was adjusted to an appropriate level, 100 μL of a cell suspension (HepG2: 4 × 10^4^ cells/mL; Hela: 4 × 10^4^ cells/mL; A549: 6 × 10^4^ cells/mL), was plated in 96-well plates. Cells were left for 6 h to attach, and medium was replaced with litchi polysaccharides which were dissolved in RPMI-1640 medium, and cells were kept at 37 °C in 5% CO_2_ for another 24 h. Then, the medium was aspirated and each well was gently rinsed with phosphate-buffered saline (PBS) twice. Cells were stained and fixed by adding 50 μL methylene blue solution (HBSS + 1.25% glutaraldehyde + 0.6% methylene blue) to each well. After 1 h incubation, plates were rinsed by gently submerging in distilled water six times. Plates were drained and air-dried before addition of 100 μL elution solution (50% ethanol + 49% PBS + 1% acetic acid) to each well and homogenized for 15 min to fully dissolve the stained materials. The plates were read using a microplate reader at 570 nm.

#### 3.4.2. Inhibition Assay of Tumor Cells Proliferation

The inhibition effects of the polysaccharides on the growth of tumor cells were evaluated by a methylene blue method described previously. HepG2, Hela and A549 cells were plated at the density of 2 × 10^4^ cells/mL, 2 × 10^4^ cells/mL and 4 × 10^4^ cells/mL, respectively, in 96-well plates and left for 6 h to attach. Then, litchi polysaccharides containing medium was added to replace the old medium at final concentrations of 50, 150, 300, 450, 600, and 750 μg/mL. After 72 h of incubation, cells were stained with methylene blue solution for 1 h. Cells were then rinsed with water and dried. Methylene blue stain was eluted with the elution solution by agitating plates at room temperature for 1 h. The absorbance was measured at 570 nm by a microplate reader (Thermo Labsystems, Helsinki, Finland). The inhibition rate (%) was calculated as: (1 − ODs/ODc) × 100, where ODs and ODc represented the OD value of the samples and control group, respectively.

### 3.5. In Vitro Immunostimulatory Activity Assay of Litchi Polysaccharides

#### 3.5.1. Determination of Mouse Splenocyte Proliferation

The splenocyte proliferation assay was performed according to a reported method [28]. Briefly, the male BALB/c mice were sacrificed by cervical dislocation and their spleens were removed aseptically and minced in PBS. The splenic cells were obtained through sterilized meshes (200 mesh) at room temperature. After lysing red blood cells with hemolytic Gey’s solution, the remaining cells were washed twice and resuspended in RPMI 1640 complete medium. The overall cell viability was greater than 95% and was determined using the trypan-blue dye exclusion technique. 

One hundred microliters of splenic cell suspension was seeded in 96-well plates at 5 × 10^6^ cells/mL with litchi polysaccharides containing medium at final concentrations of 0, 50, 100, 200 and 400 μg/mL. The cells were cultured at 37 °C and 5% CO_2_ with or without 5 μg/mL ConA or 10 μg/mL LPS for 68 h. Then, MTT (20 μL, 5 mg/mL) was added to each well. After a further 4 h of incubation, 100 μL acidified isopropyl alcohol was added to dissolve the formazan crystals for 12 h at 37 °C. The plates were finally read at 570 nm in a microplate reader. The proliferation indexes (%) of splenocyte were calculated as: (ODs/ODc − 1) × 100, where ODs and ODc represented the OD value of the samples and control group, respectively.

#### 3.5.2. Cytotoxicity Assay of Natural Killer Cells

Splenocytes were prepared as the effector cells for a splenic natural killer (NK) activity assay as described by Yi *et al.* [42]. The splenocytes were plated into 96-well plates at a density of 1 × 10^7^ cells/mL per well in a 50 μL volume, and stimulated with 40 μL different concentrations of litchi polysaccharides (ultimate concentration: 0, 50, 100, 200, 400 μg/mL) for 24 h, with twelve replicate wells employed for each concentration. Then, YAC-1 cells (10 μL, 1 × 10^6^ cells/mL) were added into six wells as the experimental group and complete medium was placed in the other six wells of the twelve replicate wells as the effector control. At the same time, complete medium (100 μL), containing YAC-1 cells (10 μL, 1 × 10^6^ cells/mL) was added into empty wells as the target control. The plates were incubated for 4 h, followed by another 4 h with MTT (20 μL, 5 mg/mL). Then, acidified isopropyl alcohol (100 μL) was added to each well followed by a 12 h incubation. The absorbance was measured at 570 nm using a microplate reader. Cytotoxicity of NK cell was expressed as percent lysis of target cells: [OD_T_ − (ODexp − ODE)]/OD_T_ × 100, where OD_T_, ODexp and OD_E_ represented the OD value of the target control, experimental group and effector control group, respectively.

#### 3.5.3. Assay of Phagocytosis of RAW264.7 Macrophages

RAW264.7 macrophages (1 × 10^5^ cells/well) were seeded in 96-well plates and allowed to adhere for 6 h. After removing the growth media from each well, different concentrations of litchi polysaccharides (0, 50, 100, 200, 400 μg/mL) prepared with culture medium, were added to each well in 100 μL and 10 μg/mL LPS was used as a positive control. After 24 h of incubation, the supernatant was removed and each well was gently rinsed with PBS twice. One hundred microliters of 0.075% neutral red solution was added to each well and the plates were cultured for a further 4 h. After washing out unphagocytized neutral red with PBS, cell lysis buffer (100 μL, acetic acid/ethanol = 1:1, mL/mL) was added to each well for 12 h. The OD value of each well was measured at 570 nm. The phagocytosis index (%) was calculated as: (OD_S_/OD_C_ − 1) × 100, where OD_S_ and OD_C_ represented the OD values of the stimulated group and control group, respectively. 

### 3.6. Statistical Analysis

Data were expressed as the mean ± standard deviations (SD). The significance of the difference was evaluated with one-way ANOVA followed by the Student-Newman-Keuls test using SPSS 19.0 software and a *p*-value of 0.05 was used as the threshold for significance. The statistically significant differences between the two groups were evaluated with an independent-sample *T*-test.

## 4. Conclusions

The drying process yielded polysaccharides with more uronic acid and protein as well as lower overall molecular weight than those obtained from fresh litchi. The monosaccharide molar ratio of LPF and LPD were significantly different. LPD showed relatively higher cytotoxic activity against three different tumor cell lines and *in vitro* immunomodulatory activity than LPF. Therefore, hot air drying can effectively change the chemical composition of litchi polysaccharides and improve their bioactivity. This finding supports the application of dried litchi pulp as a Traditional Chinese Medicine. Furthermore, the structural and bioactivity differences observed for polysaccharides obtained from dried litchi using different methods is an ongoing topic of research.
